# Forecasting New Tuberculosis Cases in Malaysia: A Time-Series Study Using the Autoregressive Integrated Moving Average (ARIMA) Model

**DOI:** 10.7759/cureus.44676

**Published:** 2023-09-04

**Authors:** Mohd Ariff Ab Rashid, Rafdzah Ahmad Zaki, Wan Rozita Wan Mahiyuddin, Abqariyah Yahya

**Affiliations:** 1 Department of Social and Preventive Medicine, Faculty of Medicine, Universiti Malaya, Kuala Lumpur, MYS; 2 Environmental Health Research Centre, Institute for Medical Research, Kuala Lumpur, MYS

**Keywords:** time series, early warning surveillance, sarima model, malaysia, tuberculosis

## Abstract

Background

The application of the Box-Jenkins autoregressive integrated moving average (ARIMA) model has been widely employed in predicting cases of infectious diseases. It has shown a positive impact on public health early warning surveillance due to its capability in producing reliable forecasting values. This study aimed to develop a prediction model for new tuberculosis (TB) cases using time-series data from January 2013 to December 2018 in Malaysia and to forecast monthly new TB cases for 2019.

Materials and methods

The ARIMA model was executed using data gathered between January 2013 and December 2018 in Malaysia. Subsequently, the well-fitted model was employed to make projections for new TB cases in the year 2019. To assess the efficacy of the model, two key metrics were utilized: the mean absolute percentage error (MAPE) and stationary R-squared. Furthermore, the sufficiency of the model was validated via the Ljung-Box test.

Results

The results of this study revealed that the ARIMA (2,1,1)(0,1,0)_12_ model proved to be the most suitable choice, exhibiting the lowest MAPE value of 6.762. The new TB cases showed a clear seasonality with two peaks occurring in March and December. The proportion of variance explained by the model was 55.8% with a p-value (Ljung-Box test) of 0.356.

Conclusions

The application of the ARIMA model has developed a simple, precise, and low-cost forecasting model that provides a warning six months in advance for monitoring the TB epidemic in Malaysia, which exhibits a seasonal pattern.

## Introduction

As per the WHO Global Tuberculosis (TB) Report of 2022, approximately 10.6 million new TB cases were diagnosed worldwide in the year 2021 [1]. Within the same year, 1.6 million people died from TB. However, this number only represents 70% of all TB cases due to unreported and undiagnosed cases. The complexity of TB situations persists due to multiple factors, including the rising incidence of human immunocompromised virus (HIV) infections, antimicrobial resistance cases, the influx of immigrants from countries with high TB burdens, the presence of non-communicable diseases, and international travel patterns [2]. In 2021, the largest proportion of individuals who were affected by TB were concentrated in Southeast Asia, accounting for 45% of the cases based on geographical distribution. Derived from information gathered from the Ministry of Health Malaysia (MOH), the number of TB cases in Malaysia has remained high for the past 30 years despite high cure rates that are achievable with a timely diagnosis and an appropriate antibiotic treatment. Regarding the burden of TB, Malaysia falls into the intermediate category, with an estimated total TB incidence of 97 (with a range of 79 to 106) per 100,000 population in the year 2021 [3]. Throughout the past few decades, the containment of TB has been an integral component of the strategic agenda outlined by the MOH Malaysia [4]. Accurately projecting its repercussions holds significant importance in formulating a well-informed strategy for an effective response [5].

The Box-Jenkins autoregressive integrated moving average (ARIMA) method is a time-series forecasting technique that relies on the behavior of observed variable data. Its strength lies in its ability to handle diverse data patterns, requiring the data to be stationary initially. Furthermore, it is highly proficient in accurate short-term forecasting. Lately, the ARIMA model has gained substantial traction in the domain of forecasting infectious disease cases, emerging as the predominant methodology for TB prediction worldwide [6-9]. It has shown a positive impact on public health early warning surveillance due to its capability in producing reliable forecasting values [10-11]. For example, in recent studies conducted in Iran and China, the correctly forecasted values helped stakeholders on developing early warning systems to support their public health prevention and intervention program in the future [12-13]. In their efforts to predict future events, researchers have primarily relied on TB databases sourced from the MOH to create predictive models. Currently, a majority of these models exhibit a significant level of complexity due to their reliance on extensive databases containing a variety of different variables. Gathering such data always comes with a cost, which can lead to a shortage of appropriate datasets for model formulation. An example of a statistical modeling approach in time-series analysis is the ARIMA model, which demands fewer data points compared to the deterministic simulation methodology. Numerous studies have suggested that time-series modeling is more suitable than the simplistic trend fitting approach, even though the former can be susceptible to model specification errors in its trend component [14]. To address this, Box and Jenkins adeptly compiled pertinent information in a comprehensive manner, enabling users to comprehend and implement this time-series model effectively [15]. In addition, the model methodology for predicting TB cases can be readily adopted by individuals without expertise, making it accessible to non-experts as well.

In Malaysia, there remains a knowledge gap regarding the creation of forecasting models, specifically for the TB disease. Predicting TB cases in Malaysia holds great importance as it offers a vital foundation for public health planning and intervention initiatives, crucial in addressing the TB epidemic. Studies focused on TB and employing forecasting models have predominantly been confined to local areas, with the comprehensive nationwide trend yet to be documented [16-17]. To bridge this knowledge gap, the current study seeks to explore the effectiveness of utilizing a time-series analysis, specifically the ARIMA approach, to predict new TB cases in Malaysia. This will be accomplished by utilizing TB data from 2013 to 2018. The resultant model will subsequently be applied to forecast new TB cases for the year 2019. As a result, this study seeks to improve the effectiveness of the TB surveillance program, thereby contributing to the management and control of TB outbreaks.

## Materials and methods

 Study area

The study was carried out in Malaysia, an Asian nation situated in Southeast Asia. Malaysia is partitioned by the South China Sea into two main regions: Peninsular Malaysia, encompassing 11 states and two federal territories, and East Malaysia, consisting of two states and one federal territory. Kuala Lumpur serves as the capital city, and Putrajaya acts as the hosting location for the federal government. Sharing borders with Thailand and Singapore, Malaysia spans an area of roughly 330,803 square kilometers and maintains a population density of approximately 92 individuals per square kilometer. According to the 2019 report from the Department of Statistics, Malaysia's total population had risen to 32.4 million. Healthcare in Malaysia is primarily subsidized by the government's MOH, operating within a two-tiered system that encompasses both government and private healthcare systems. An integral national health priority involves the reduction of infectious disease burdens, including TB. The changing patterns of TB contribute to some of the central health challenges faced by the nation.

Study data source

The dependent variable is the new TB cases, which are newly registered cases in the TB Information System (TBIS). These cases are determined through chest physician evaluations, laboratory tests, and radiography findings. All data span from January 2013 to December 2018. Data from January 2013 to June 2018 were covered for model fitting, and the remaining six months of data (July 2018 to December 2018) were used for model prediction. Throughout the study period, Malaysia consistently executed stable TB control programs on an annual basis.

Ethical approval was obtained from the National Medical Research Registry (NMRR) NMRR-19-451-45857 (IIR). The analysis was primarily conducted using IBM SPSS Statistics for Windows, version 23.0 (released 2015, IBM Corp., Armonk, New York, United States) to develop the ARIMA model.

Time-series analysis

During the study, an ARIMA model was developed using the time-series data of new TB cases. The initial stage in employing the Box-Jenkins methodology involves the identification of the appropriate ARIMA class for the given data series. Autocorrelation function (ACF) and partial autocorrelation function (PACF) coefficients are common statistics used for this purpose. ARIMA models are categorized as ARIMA (p, d, q)(P, D, Q)s. In the case of non-seasonal ARIMA, the parameters include autoregressive terms (p), differencing orders (d), and moving average terms (q). For seasonal ARIMA (P, D, Q) or SARIMA, the model incorporates seasonal autoregressive terms (P), seasonal differencing (D), and seasonal moving average terms (Q), all of which are determined in relation to a specific period denoted as "s." The methodology follows the foundational steps of the Box-Jenkins approach for time-series modeling, which consists of three phases: identification, estimation/testing, and application. During the identification phase, the assessment of the dependent variable's stationarity involves the examination of ACF and PACF with a lag equivalent to one year (12 months). ARIMA requires the dependent variable to be stationary, which means a constant mean and variance over time. Stationarity can be attained by either applying differencing to the series or utilizing a logarithmic conversion on the dependent variables [18].

In this research, the stationarity of the series was achieved through both seasonal and nonseasonal differencing. The examination of correlograms indicated that the series became stationary, with residuals showing no time-based correlation, and fluctuating around a constant mean with zero variance. Once the data have been made stationary, the subsequent step involves generating ACF and PACF plots. These plots serve as the foundation for constructing the preliminary ARIMA prediction model (p, d, q), where the value of order "p" is ascertained from the PACF plot and the value of order "q" is derived from the ACF plot. The determination of order "d" is contingent on the number of differencing operations applied to the data. The most suitable model was identified by comparing the mean absolute percentage error (MAPE) and the stationary R-squared value. Lower MAPE values were favored, while a higher stationary R-squared value indicated a larger proportion of the dependent variable's variance explained by the model. Once the optimal model was identified, forecasts were generated for monthly values spanning January to December 2019.

## Results

Explanatory statistics

**Figure 1:**
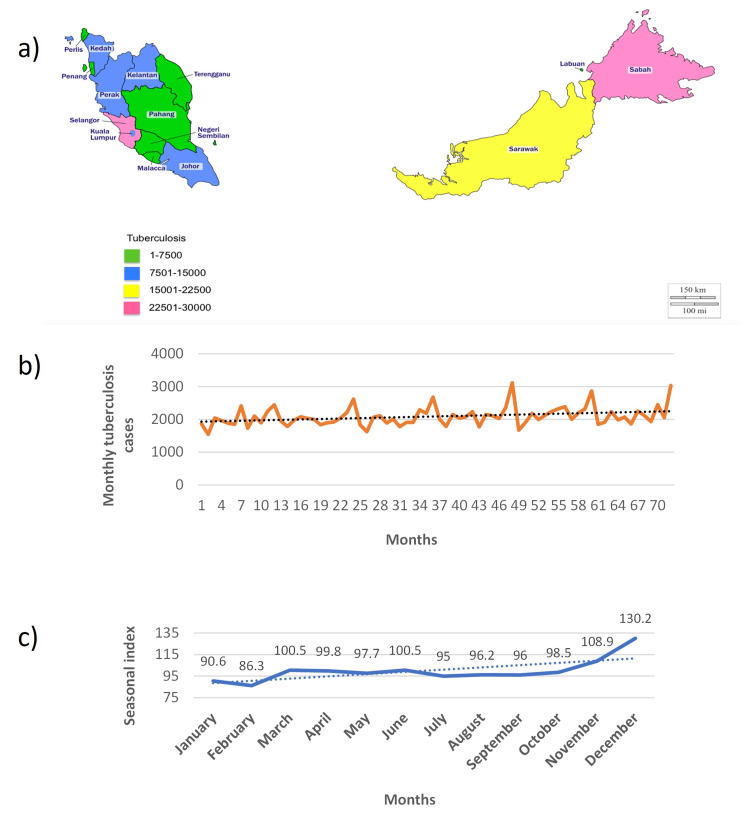
(a) Geographical distribution of new TB cases in Malaysia from January 2013 to December 2018; (b) trend analysis of new TB cases in Malaysia from January 2013 to December 2018; (c) annual cycle of new TB cases in Malaysia from January 2013 to December 2018. Figure 1 is the original work of the authors.

The total number of new TB cases in Malaysia from January 2013 to December 2018 was 150,606 cases. It was found that the new tuberculosis cases showed an uptrend from 24,007 new cases in 2013 to 25,900 new cases in 2018. The maximum and minimum new TB cases were 3,166 cases/month and 1,545 cases/month, respectively. The monthly average of new TB cases from January 2013 to December 2018 was 2,092 cases (95% confidence interval (CI) 2022-2162). Based on data from the TBIS from January 2013 to December 2018, the highest number of new TB cases was recorded in Sabah with a total number of 28,802 cases. The other states with high new TB cases were Selangor (27,868) and Sarawak (16,788). The lowest number of new TB cases was recorded in Labuan and Perlis with a total number of 712 and 794 cases, respectively. Throughout these years, three states, namely, Selangor, Sabah, and Sarawak, had shown the highest increment of new TB cases compared to other states in Malaysia. The distribution of new TB cases by states in Malaysia from 2013 to 2018 is shown in Figure 1a.

Time-series analysis

Figure 1b shows the time-series sequence plot of monthly new TB cases from January 2013 to December 2018 in Malaysia. Over the study duration, there was variability in the monthly new tuberculosis cases across the months, following a positive upward trajectory depicted by the trend equation *y* = 1925.45 × time + 4.506. Hence, in our further modeling strategy, we assumed that the number of new TB cases fluctuates around a consistent mean. Furthermore, the annual cycle of a seasonal index of the new TB cases exhibited a seasonal pattern from January 2013 to December 2018, as shown in Figure 1c. The highest seasonal index of new TB cases was recorded in December, November, and then March. Meanwhile, the lowest was recorded in February. This was consistent with the findings in Figure 1c, in which a seasonal pattern was observed at two periods of time. The number of new TB cases started to increase in February until March before gradually decreasing to the end of September. The new TB cases then increased again in October and then reached the peak point in December. Furthermore, the seasonal index ranged from the lowest of 86.3 in February to 130.2 in December, which suggested that the seasonal swing in a complete cycle of year was from 86.3% on average to 130.2% on average.

**Figure 2:**
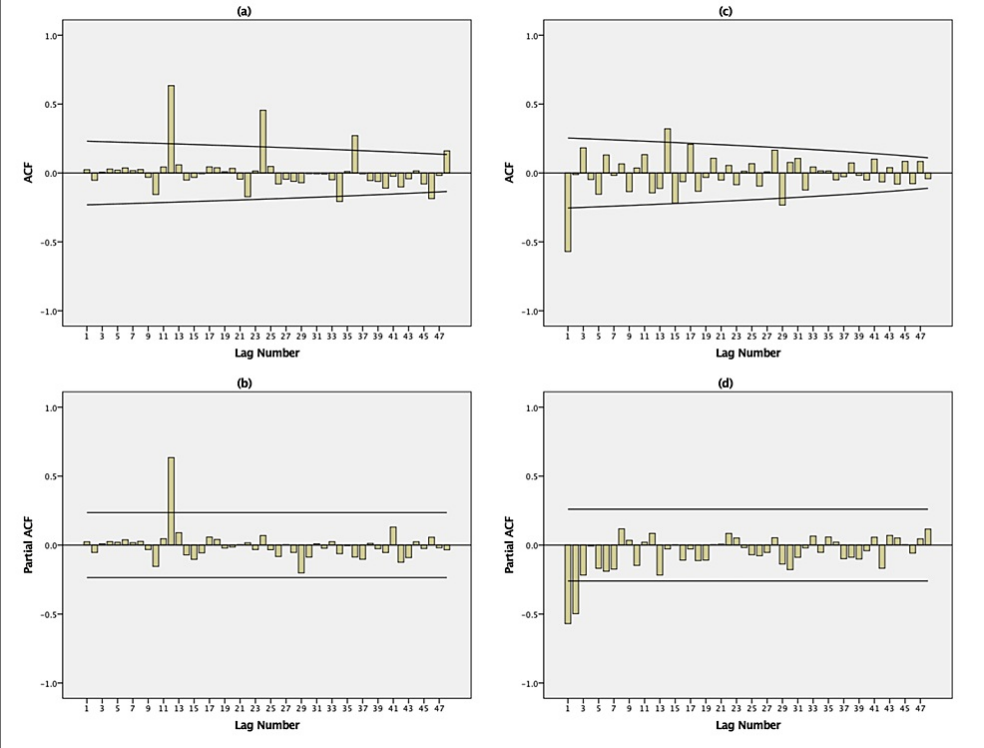
Autocorrelation function (ACF) and partial autocorrelation function (PACF) of data and transformed series footnote: (a) ACF without any difference; (b) PACF without any difference; (c) ACF with both seasonal (1, period 12) and nonseasonal difference (1); (d) PACF with both seasonal (1, period 12) and nonseasonal difference (1). The lag number refers to every 12-month interval (lags 1-12) at lags 12, 24, 36, and 48. With 72 monthly (January 2013-December 2018) values used for the model synthesis, correlations at the first 48 lags were examined. Figure 2 is the original work of the authors.

The results of the ACF and PACF examinations for new TB cases unveiled that the series lacked stationarity, primarily due to the presence of seasonal components (Figure 2a, 2b). The ACF plot of seasonality in Figure 2a exhibits strong positive autocorrelation at several times at lags 12, 24, and 36 (lags 12, 24, and 36 represent the month of December). From Figure 2b, it can be observed that the PACF graph representing seasonality displayed a notable peak at a lag of 12 (corresponding to the month of December). This peak serves as a confirmation for the existence of the annual seasonal element within the series. Hence, to achieve stationarity in the series, we conducted first-order differencing adjustments both seasonally and nonseasonally. As a result, an ARIMA (p,1,q)(P,1,Q)_12_ model was chosen as the fundamental framework for the potential model.

Figures 2c and 2d show the stationary behavior of the series, with uncorrelated residuals over time and consistent fluctuations around a constant mean and zero variance. The ACF plot in Figure 2c implies a moving-average order of *q* being 1, as the autocovariance approach zero for lags beyond 1. Meanwhile, the post-differencing PACF plot in Figure 2d indicates that the value of *p* should be 2, as the partial autocorrelations are in proximity to zero for all lags surpassing 2.

**Figure 3:**
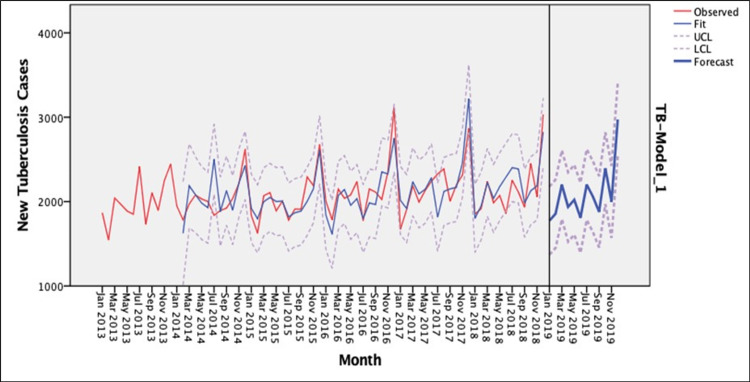
Observed and forecasted monthly new TB cases in Malaysia for 2013-2019. The red graph (observed) refers to the actual number of new TB cases for the period of January 2013-December 2018. The light-blue graph (fit) refers to the model-fitted values/number of new TB cases for the period of January 2013-December 2018. The dotted graph (upper confidence level (UCL) and lower confidence level (LCL)) refers to the upper and lower 95% confidence intervals of new TB cases for the period of January 2013-December 2018. The thick blue (forecast) refers to the forecasted number of new TB cases for the period of January-December 2019. Figure 3 is the original work of the authors.

Table 1 presents the MAPE, stationary R-squared, and p-values for the various ARIMA models associated with different choices of *p*, *d*, and *q*. The optimal model was determined by evaluating the model fit statistics and the Ljung-Box test. None of the models were statistically significant (p > 0.05), indicating no significant autocorrelation between residuals at different lag times, thereby suggesting that the residuals represented white noise. The best model was the model with the lowest value of MAPE and higher stationary R-squared value. The ARIMA model (2,1,1)(0,1,0)_12_ emerged as the best selection, boasting the lowest MAPE of 6,762 (as presented in Table 1). This model accounted for 55.8% of the variance, and its adequacy was validated through the Ljung-Box test with a p-value of 0.356. The forecasts generated by this model closely aligned with the observed values of the monthly mean for new TB cases, exhibiting an MAPE of approximately 6%. Consequently, the ARIMA (2,1,1)(0,1,0)_12_ model was deemed the optimal choice within the ARIMA methodology, aligning with the principle of parsimonious modeling. The ARIMA (2,1,1)(0,1,0)_12_ model was developed using data collected from January 2013 to June 2018, and its accuracy was assessed using data gathered between July and December 2018. The model's validity was tested and then utilized to produce forecasts for new TB cases spanning January to December 2019. The outcomes demonstrated a satisfactory alignment between the actual values and predicted values, as depicted in Figure 3. According to the new TB cases prediction (Figure 3), a distinct seasonal trend was observed, showing a notable increase in new TB cases across the January-December 2023 timeframe in Malaysia. 

**Table 1:** Model fit statistics of the proposed ARIMA models based on the MAPE and stationary R-squared values and p-value (Ljung-Box test). Table 1 is the original work of the authors. ARIMA, autoregressive integrated moving average; MAPE, mean absolute percentage error

	Model fit statistics		Ljung-Box test
Models	MAPE	Stationary R-squared	Ljung Box p-value (at 5% significance level)
ARIMA (0,1,1)(0,1,0)_12_	7.204	0.539	0.158
ARIMA (1,1,1)(0,1,0)_12_	6.806	0.554	0.207
ARIMA (2,1,0)(0,1,0)_12_	7.378	0.500	0.152
ARIMA (2,1,1)(0,1,0)_12_	6.762	0.558	0.356

## Discussion

An early epidemic prediction tool plays a crucial role in assessing outbreak risks. Detecting outbreaks early allows for timely interventions and prevention measures rather than just managing a full-blown epidemic. The necessity for an early warning system arises to pinpoint and assess the risk of TB within a population. The ARIMA model proves valuable for interpreting and applying surveillance data. Its potential is significant as a decision support tool, aiding in enhancing preparedness planning and public health interventions in the field. The findings of this study demonstrate that the developed ARIMA model closely mirrors the trend of new TB cases, confirming the presence of TB in Malaysia. This predictive model was constructed using data on new TB cases recorded from January 2013 to December 2018. It assumes a sustained pattern in various conditions, such as climatic factors, control measures, prevention efforts, treatment-seeking behavior, and population migration. The model's validation process established its suitability for Malaysia's context, evidenced by the manageable level of errors in this forecasting study.

Among all models, ARIMA (2,1,1)(0,1,0)_12_ was found as the best-fit predictive model in our study, with the least MAPE values and highest stationary R-squared. The result predicted that the number of new cases of TB in Malaysia from January to December 2019 was 1,899, 1,950, 2,217, 1,984, 2,113, 1,872, 2,236, 2,124, 2,964, 2,452, 2,047, and 3,049 cases, respectively. These 12-month forecasted new TB cases will be a useful tool for the MOH Malaysia to estimate the real number of new TB cases in Malaysia while targeting the control measures at the states using the spatial distribution of new TB cases of the previous years. The results in this study also matched several studies in other Asian countries, which predicted seasonal and increasing patterns in new TB cases. In Iran, a study identified the ARIMA (0,1,1)(0,1,1)_12_ model as the best fit for TB data, with the peak cases occurring in May [12]. Meanwhile, a study in China found that the ARIMA (1,0,0)(0,1,1)_12_ model was deemed best fit with seasonal differencing [13]. However, different ARIMA models were found for different countries, which suggests that each country has its own trend. The differences in TB trends between countries were mainly due to variation in control measures, patients' health-seeking behaviors, and climatic factors. Other factors, such as increased number of HIV infections and immigrants, may also have partly contributed to the number of TB cases in each country.

In this study, we also recognized a seasonal distribution based on the monthly new TB cases from January 2013 to December 2018 in Malaysia. From an overall perspective, the monthly new TB cases was peaked in March with a trough in December. This is also consistent with the predicted data for 2019 with regard to the timing of the peak. Similar patterns in the seasonal distribution of new TB cases have also been observed in other studies, including those conducted in Malaysia. For instance, Portugal also exhibited a peak in new TB cases during March, followed by a trough in December [19]. Meanwhile, across the entirety of the United States, the highest tuberculosis case count was also observed in March, whereas the lowest point occurred in November [20]. The new disease spread and increased cases of latent infections were usually the main factors that lead to the peak of TB cases. Therefore, whenever a study on the peak seasonal TB cases is performed, factors, such as delays in diagnosis, should be taken into account [21]. A prior study indicated that the median delay for TB patients was 93 days, with a range spanning from 68 to 128 days [22]. Failure to achieve early diagnosis combined with the variability in TB's incubation periods results not only in an extended infectious period and higher transmission rates but also undermines the dependability of the TB surveillance system and the efficacy of an early warning system [23]. Furthermore, it is important to highlight that the school and festival holidays in Malaysia lead to an elevated number of new TB cases in March and December, primarily due to the movement of the population. Further research is advised to integrate the forecasting model into the current disease control program. This involves discussing preventive measures, monitoring protocols, and compliance efforts to address population mobility's impact on TB transmission during specific time periods.

It is important to recognize limitations in interpreting forecasts. Factors, such as socioeconomic conditions and natural environments, could affect TB cases, but they were not considered due to data constraints and the study's focus on time-series forecasting. The ARIMA model is best for short-term predictions, requiring frequent new data for accuracy. Regular updates and adjustments to the model are crucial for dependable disease transmission predictions. In conclusion, the ARIMA model is best suited for short-term forecasting purposes. It is important to note that this model necessitates dynamic updating with new data to ensure the accuracy and stability of its forecasts over time.

## Conclusions

The ARIMA (2,1,1)(0,1,0)_12_ model is the best forecasting model for new TB cases in Malaysia. This model offers the MOH with advanced predictions of new TB cases, providing valuable guidance for timely planning of prevention and control measures. Having advanced knowledge into the spatial distribution of new TB cases in Malaysia for the upcoming 12 months would significantly assist in precisely directing control measures across the nation.
